# Supplementary material for: Physiochemical interaction between osmotic stress and a bacterial exometabolite promotes plant disease
Source: Nat Commun. 2024 May 28;15:4438. doi: 10.1038/s41467-024-48517-5 (PMC11133316; doi:10.1038/s41467-024-48517-5)
Supplement: Supplementary file 1 — Supplementary Information [file 41467_2024_48517_MOESM1_ESM.pdf]

Supplementary information for

Physiochemical interaction between osmotic stress and a bacterial exometabolite promotes plant disease

Felix Getzke[1,#], Lei Wang[2,#], Guillaume Chesneau[1], Nils Böhringer[2,3], Fantin Mesny[1,6], Nienke Denissen[1], Hidde Wesseler[1], Priscilla Tijesuni Adisa[1], Michael Marner[4], Paul Schulze-Lefert[1,5], Till F. Schäberle[2,3,4,*], Stéphane Hacquard[1,5,*]

[1]Department of Plant Microbe Interactions, Max Planck Institute for Plant Breeding Research, 50829 Cologne, Germany. [2]Institute for Insect Biotechnology, Justus-Liebig-University Giessen, 35392 Giessen, Germany. [3]German Center for Infection Research (DZIF), Partner Site Giessen-Marburg-Langen, 35392 Giessen, Germany. [4]Fraunhofer Institute for Molecular Biology and Applied Ecology (IME), Branch for Bioresources, 35392 Giessen, Germany. [5]Cluster of Excellence on Plant Sciences (CEPLAS), Max Planck Institute for Plant Breeding Research, 50829 Cologne, Germany. [6]Present address: Institute for Plant Sciences, University of Cologne, 50674 Cologne, Germany. [#]contributed equally to this work.

*Correspondence: Stéphane Hacquard: hacquard@mpipz.mpg.de and Till F. Schäberle: Till.F.Schaeberle@agrar.uni-giessen.de

Supplementary figures

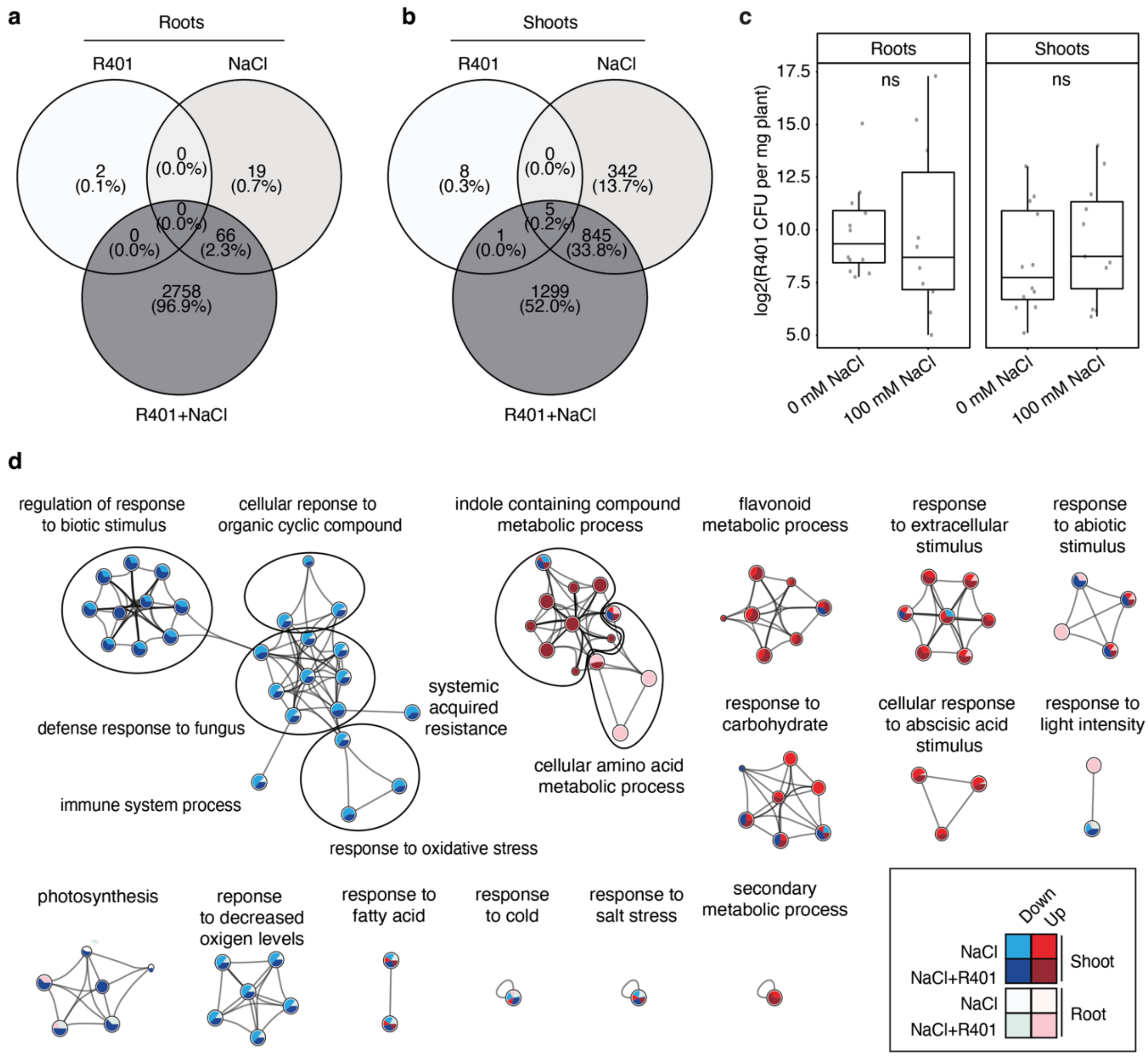

Supplementary Fig. 1: R401 colonisation and transcriptome modulation are not linked. (**a**,**b**) Venn diagram of DEGs (from **Fig. 2a**) in roots (**a**) and shoots (**b**) of *A. thaliana* plants co-inoculated with R401, 100 mM NaCl or both in the gnotobiotic Flowpot system. DEGs derive from a pairwise comparison to control (HK and 0 mM NaCl)-treated plants. Percentages indicate the total number of DEGs of all three comparisons**.** (**c**) Colonization capability of R401 on roots and shoots of *A. thaliana* seedlings grown in the gnotobiotic Flowpot system for 28 dpi in the presence or absence of 100 mM NaCl. Colony forming units have been normalized to tissue fresh weight; n = 12 biologically independent samples each comprising 5 roots. No statistically significant differences were determined by Kruskal-Wallis followed by Dunn's post-hoc test and Benjamini-Hochberg adjustment as indicated by "ns", not significant. Boxplots show 25th percentile, median, and 75th percentile. (**d**) Network of significantly enriched GO terms with NaCl or NaCl + R401-treated shoots and roots. All significantly up- and downregulated genes as compared to control conditions (0 mM NaCl + HK R401) were selected for the analysis. GO term enrichment analysis and clustering was performed by Metascape[36]. Unfilled spheres indicate subclusters.

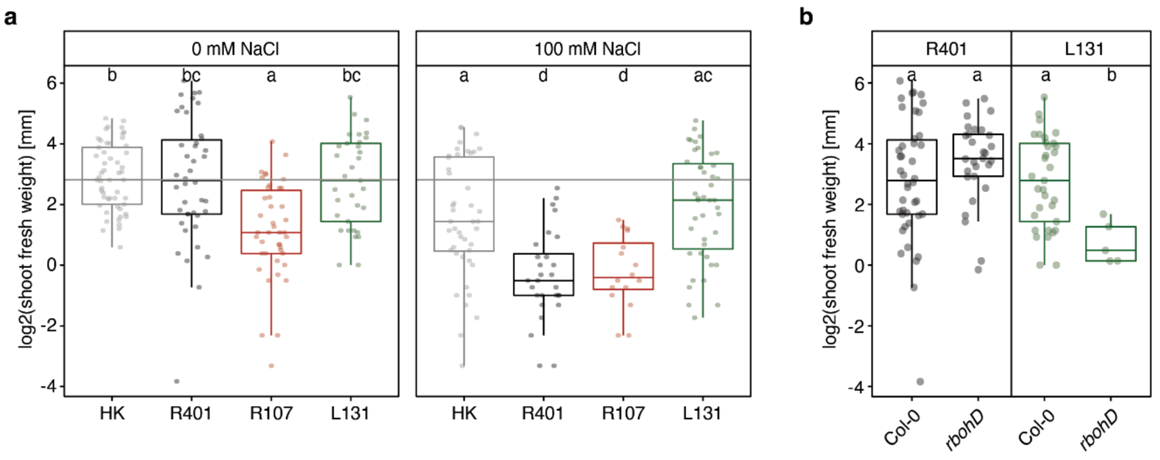

Supplementary Fig. 2: R401 does not become detrimental in an immunocompromised *rbohD A. thaliana* mutant. (**a**) log2-transformed shoot fresh weight of *A. thaliana* plants grown in the gnotobiotic Flowpot system for 28 dpi in the presence or absence of 100 mM NaCl and either heat-killed (HK) R401 cells or live R401, *Streptomyces sp.* R107 or *Xanthomonas sp.* L131 cells. (**b**) log2-transformed shoot fresh weight of *A. thaliana* wildtype (Columbia-0, Col-0) or *rbohD* mutant plants grown in the gnotobiotic Flowpot system for 28 dpi in the presence of either live R401 or *Xanthomonas sp.* L131 cells. Col-0 data are identical with (a). (**a**,**b**) Letters indicate statistically significant differences as determined by Kruskal-Wallis followed by Dunn's post-hoc test and Benjamini-Hochberg adjustment with $p < 0.05$ (n = 30 plants). Boxplots show 25th percentile, median, and 75th percentile.

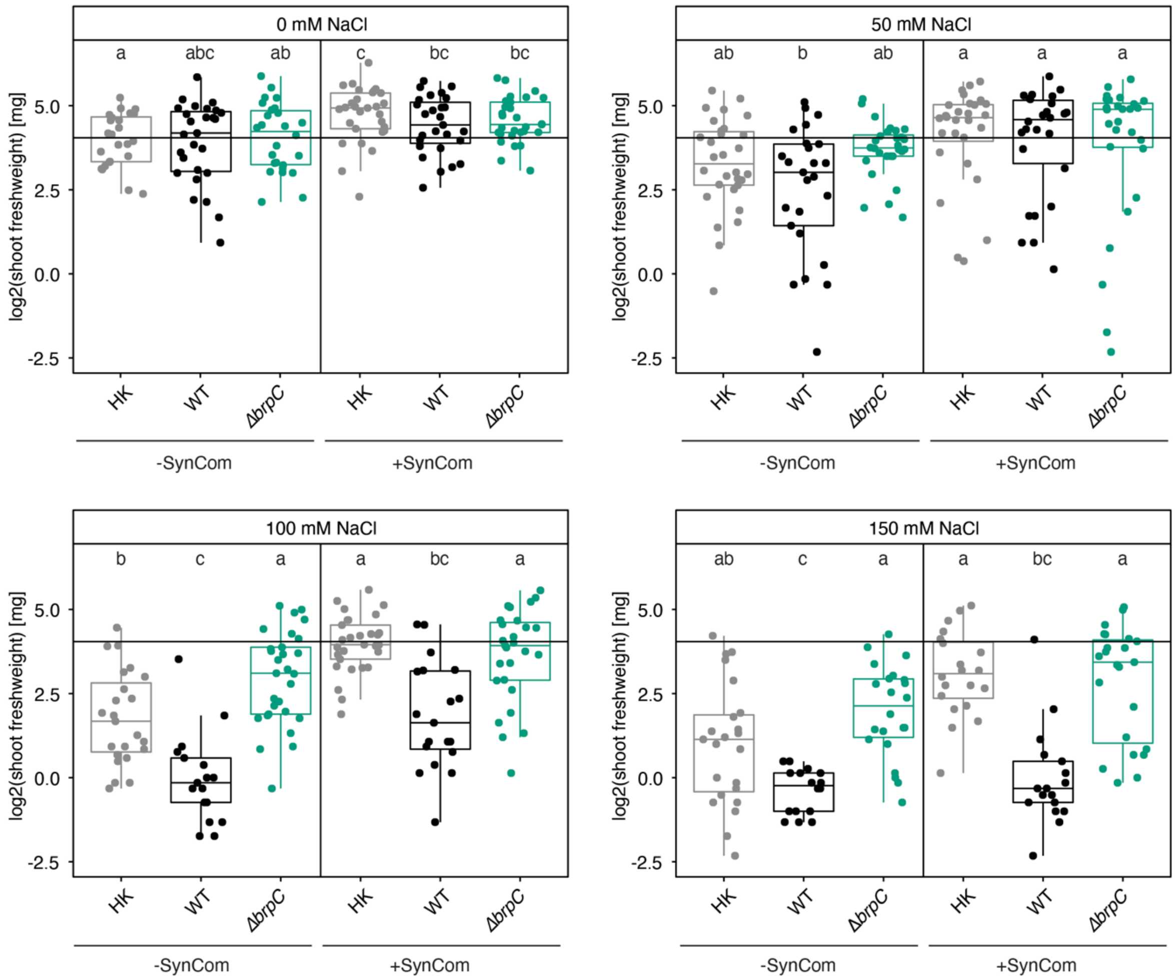

Supplementary Fig. 3: *brpC*-dependent detrimental activity of R401 is retained in a microbial community context. Log2-transformed shoot fresh weight of *A. thaliana* plants grown in the gnotobiotic Flowpot system for 28 dpi in the presence of increasing concentrations of NaCl (0, 50, 100, or 150 mM NaCl) and either heat-killed (HK), live wild-type (WT) or live Δ*brpC* R401 cells co-inoculated in the absence (-SynCom) or presence (+SynCom) of a 15-member microbial synthetic community. Letters indicate statistically significant differences as determined by Kruskal-Wallis followed by Dunn's post-hoc test and Benjamini-Hochberg adjustment with $p < 0.05$ (n = 30 plants). Statistical comparisons were conducted for each salt treatment separately. Boxplots show 25th percentile, median, and 75th percentile.

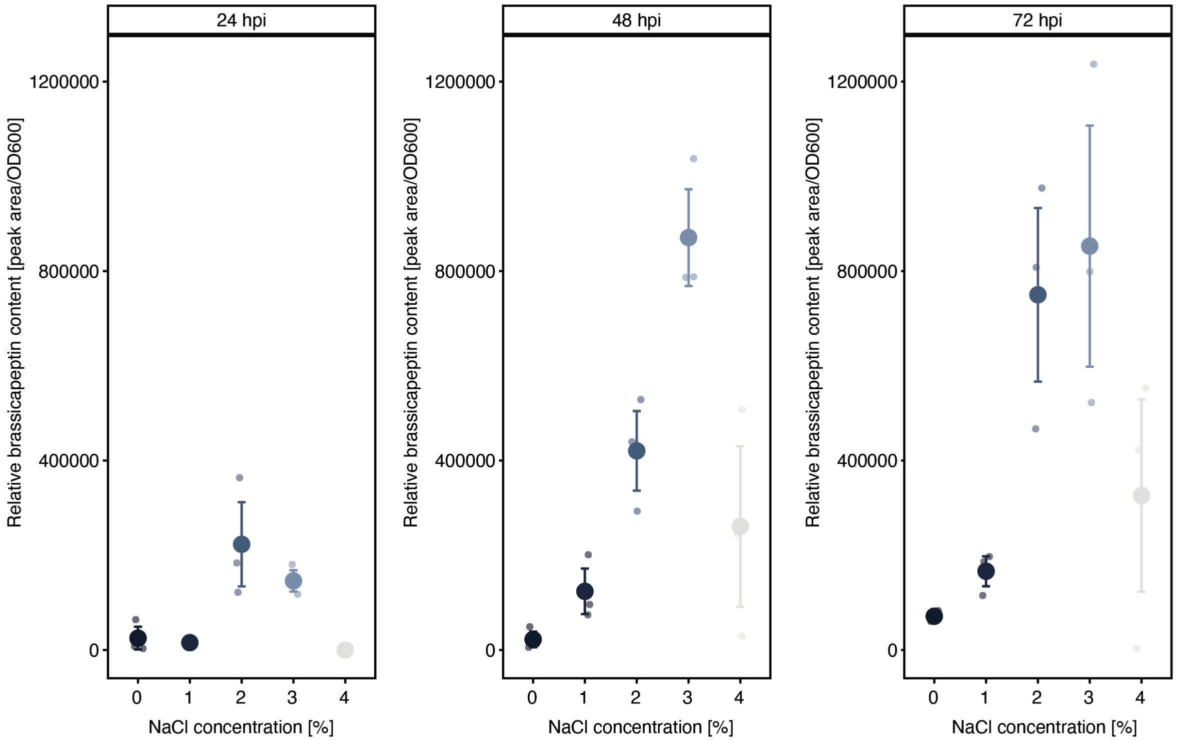

Supplementary Fig. 4: Brassicapeptin production in R401 is salt-inducible. Brassicapeptin ion abundance (peak area) relative to OD_{600} of cultures in 50 % TSB supplemented with 0 % NaCl (0 mM), 1 % NaCl (171 mM), 2 % NaCl (342 mM), 3 % NaCl (513 mM) and 4 % NaCl (684 mM) over the course of 3 days. Addition of 5 % NaCl to the growth medium did not allow any growth of *Pseudomonas* R401 (data not shown). Cultivation and analysis was performed in biological triplicates.

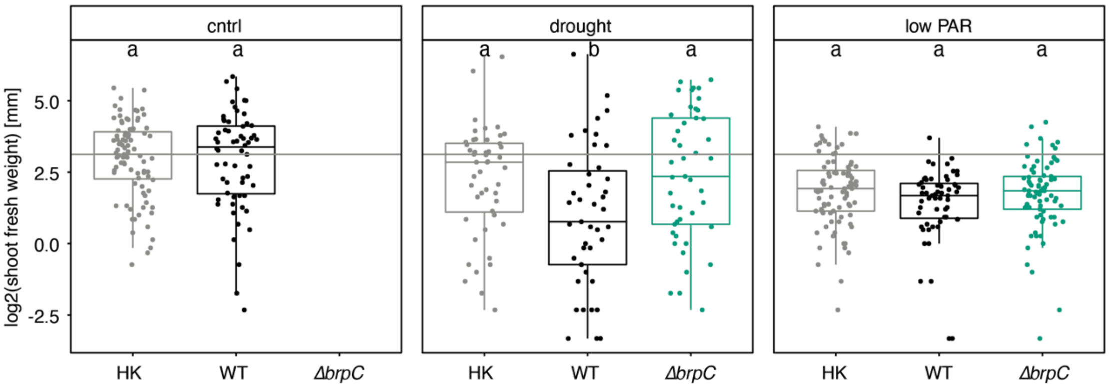

Supplementary Fig. 5: *brpC*-dependent detrimental activity of R401 on *A. thaliana* is osmotic-stress dependent. Log2-transformed shoot fresh weight of *A. thaliana* plants grown in the gnotobiotic Flowpot system for 28 dpi in the presence or absence of drought stress (5% PEG8000) or low photosynthetically active radiation (low PAR, induced by shading; Hou et al., 2021) and either heat-killed (HK), live wild-type (WT) or live Δ*brpC* R401 cells (n = 90 plants). Note that Δ*brpC* R401 was not tested in control conditions. Letters indicate statistically significant differences as determined by Kruskal-Wallis followed by Dunn's post-hoc test and Benjamini-Hochberg adjustment with $p < 0.05$.

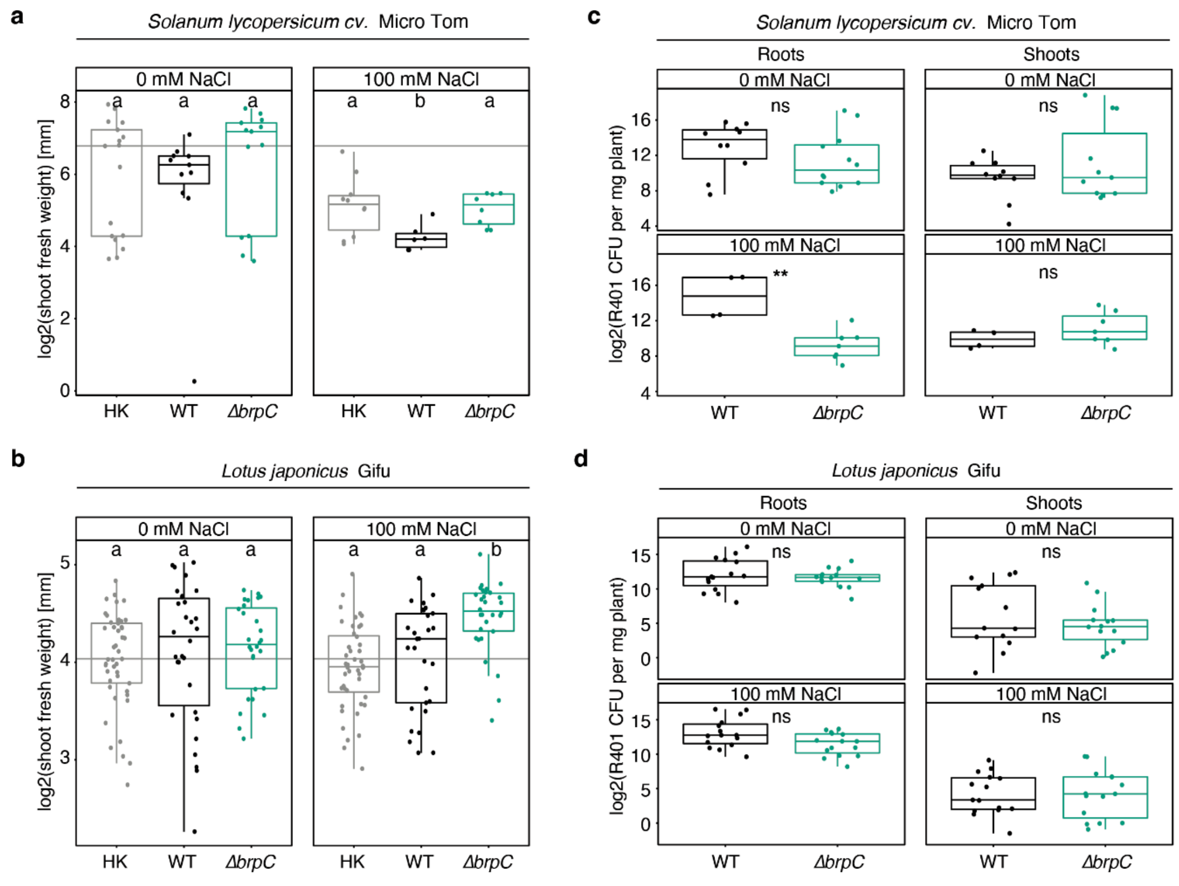

Supplementary Fig. 6: *brpC*-dependent detrimental activity of R401 is retained in salt-treated *S. lycopersicum*. (**a**,**b**) log2-transformed shoot fresh weight of *Solanum lycopersicum cv.* Micro-Tom (**a**; n = 15 plants) and *Lotus japonicus* Gifu (**b**; n = 45. plants) plants grown in the gnotobiotic Flowpot system for 28 dpi in the presence or absence of 100 mM NaCl and either heat-killed (HK), live wild-type (WT) or live Δ*brpC* R401 cells. (**a**,**b**) Letters indicate statistically significant differences as determined by Kruskal-Wallis followed by Dunn's post-hoc test and Benjamini-Hochberg adjustment with $p < 0.05$. Statistical comparisons were conducted for each abiotic stress treatment separately. (**c**,**d**) Colonization capability of R401 on roots and shoots of *Solanum lycopersicum cv.* Micro-Tom (**c**) and *Lotus japonicus* Gifu (**d**) plants grown in the gnotobiotic Flowpot system for 28 dpi in the presence or absence of 100 mM NaCl. Colony forming units have been normalized to tissue fresh weight; n = 15 samples each comprising either 1 tomato or 3 *Lotus* roots. Statistical significance was determined by Kruskal-Wallis followed by Dunn's post-hoc test and Benjamini-Hochberg adjustment. Significance between WT and Δ*brpC* is indicated by black asterisks (∗∗ indicates $p < 0.01$; ns, not significant). Statistical comparisons were conducted for each NaCl treatment and compartment separately. Boxplots show 25th percentile, median, and 75th percentile.

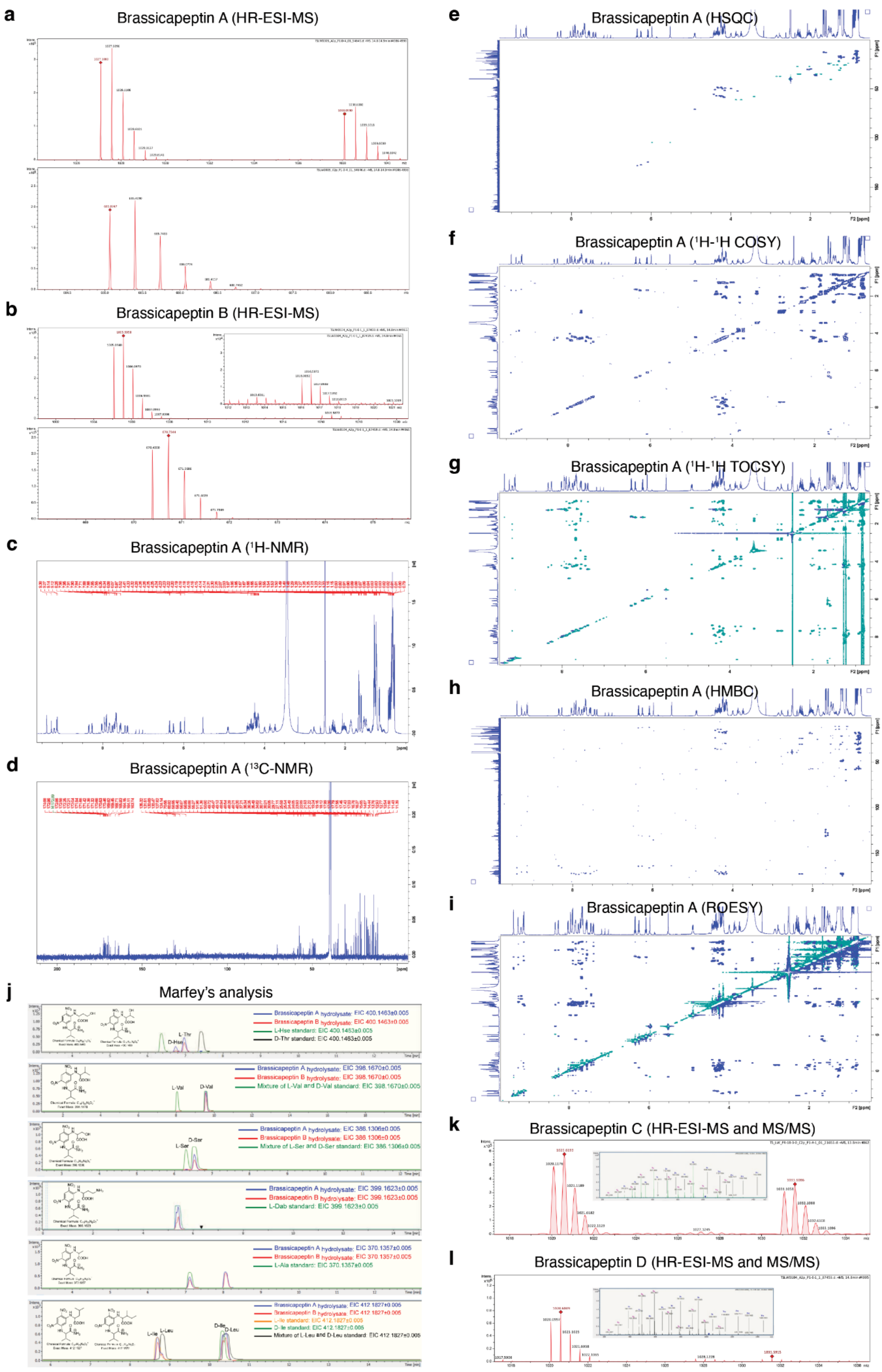
a
Brassicapeptin A (HR-ESI-MS)
b
Brassicapeptin B (HR-ESI-MS)
c
Brassicapeptin A (¹H-NMR)
d
Brassicapeptin A (¹³C-NMR)
e
Brassicapeptin A (HSQC)
f
Brassicapeptin A (¹H-¹H COSY)
g
Brassicapeptin A (¹H-¹H TOCSY)
h
Brassicapeptin A (HMBC)
i
Brassicapeptin A (ROESY)
j
Marfey's analysis
Brassicapeptin A hydrolysate: EIC 400.1463±0.005
Brassicapeptin B hydrolysate: EIC 400.1463±0.005
L-Hse standard: EIC 400.1463±0.005
D-Thr standard: EIC 400.1463±0.005
L-Thr
D-Hse
Brassicapeptin A hydrolysate: EIC 398.1670±0.005
Brassicapeptin B hydrolysate: EIC 398.1670±0.005
Mixture of L-Val and D-Val standard: EIC 398.1670±0.005
L-Val
D-Val
Brassicapeptin A hydrolysate: EIC 386.1306±0.005
Brassicapeptin B hydrolysate: EIC 386.1306±0.005
Mixture of L-Ser and D-Ser standard: EIC 386.1306±0.005
D-Ser
L-Ser
Brassicapeptin A hydrolysate: EIC 399.1623±0.005
Brassicapeptin B hydrolysate: EIC 399.1623±0.005
L-Dab standard: EIC 399.1623±0.005
Brassicapeptin A hydrolysate: EIC 370.1357±0.005
Brassicapeptin B hydrolysate: EIC 370.1357±0.005
L-Ala standard: EIC 370.1357±0.005
Brassicapeptin A hydrolysate: EIC 412.1827±0.005
Brassicapeptin B hydrolysate: EIC 412.1827±0.005
L-Ile standard: EIC 412.1827±0.005
D-Ile standard: EIC 412.1827±0.005
Mixture of L-Leu and D-Leu standard: EIC 412.1827±0.005
L-Ile
L-Leu
D-Ile
D-Leu
k
Brassicapeptin C (HR-ESI-MS and MS/MS)
l
Brassicapeptin D (HR-ESI-MS and MS/MS)

Supplementary Fig. 7: Structure elucidation of brassicapeptins. (**a**,**b**) HR-ESI-MS of brassicapeptin A (**a**) and brassicapeptin B (**b**). (**c**) ^{1}H-NMR (600 MHz, DMSO-d_6) spectrum of brassicapeptin A. (**d**) ^{13}C-NMR (150 MHz, DMSO-d_6) spectrum of brassicapeptin A. (**e**) HSQC (600 MHz, DMSO-d_6) spectrum of brassicapeptin A. (**f**) ^{1}H-^{1}H COSY (600 MHz, DMSO-d_6) spectrum of brassicapeptin A. (**g**) ^{1}H-^{1}H TOCSY (600 MHz, DMSO-d_6) spectrum of brassicapeptin A. (**h**) HMBC (600 MHz, DMSO-d_6) spectrum of brassicapeptin A. (**i**) ROESY (600 MHz, DMSO-d_6) spectrum of brassicapeptin A. (**j**) Comparison of the Marfey derivatization products of brassicapeptin A and B DCl hydrolysate and amino acid standards. (**k**) HR-ESI-MS and HR-ESI-MS/MS (insert) of brassicapeptin C. (**l**) HR-ESI-MS and HR-ESI-MS/MS (insert) of brassicapeptin D

Brassicapeptin A	$CH_3-(CH_2)_4-CO-Dhb^{1}-Ala^{2}-Ala^{3}-Leu^{4}-Ala^{5}-Val^{6}-Ile^{7}-Dhb^{8}-Hse^{9}-Val^{10}-Leu^{11}-Dha^{12}-Ala^{13}-Ala^{14}-Ala^{15}-Val^{16}-Thr^{17}-Dhb^{18}-Ala^{19}-Dab^{20}Ser^{21}-Ile^{22}$
Brassicapeptin B	$CH_3-(CH_2)_4-CO-Dhb^{1}-Ala^{2}-Ala^{3}-Leu^{4}-Ala^{5}-Val^{6}-Ile^{7}-Dhb^{8}-Gly^{9}-Val^{10}-Leu^{11}-Dha^{12}-Ala^{13}-Ala^{14}-Ala^{15}-Val^{16}-Thr^{17}-Dhb^{18}-Ala^{19}-Dab^{20}Ser^{21}-Ile^{22}$
Brassicapeptin C	$CH_3-(CH_2)_4-CO-Dhb^{1}-Ala^{2}-Ala^{3}-Leu^{4}-Ala^{5}-Val^{6}-Ile^{7}-Dhb^{8}-Hse^{9}-Val^{10}-Leu^{11}-Dha^{12}-Ala^{13}-Ala^{14}-Ala^{15}-Val^{16}-Thr^{17}-Dhb^{18}-Gly^{19}-Dab^{20}Ser^{21}-Ile^{22}$
Brassicapeptin D	$CH_3-(CH_2)_4-CO-Dhb^{1}-Gly^{2}-Ala^{3}-Leu^{4}-Ala^{5}-Val^{6}-Ile^{7}-Dhb^{8}-Hse^{9}-Val^{10}-Leu^{11}-Dha^{12}-Ala^{13}-Ala^{14}-Ala^{15}-Val^{16}-Thr^{17}-Dhb^{18}-Ala^{19}-Dab^{20}Ser^{21}-Ile^{22}$
Cichopeptin A	$CH_3-(CH_2)_n-CH=CH-(CH_2)_m-CH(OH)-CH_2-CO-Dhb^{1}-Pro^{2}-Ala^{3}-Ala^{4}-Ala^{5}-Ala^{6}-Val^{7}-Dhb^{8}-Gly^{9}-Val^{10}-Ile^{11}-Gly^{12}-Ala^{13}-Val^{14}-Ala^{15}-Val^{16}-Dhb^{17}-Thr^{18}-Ala^{19}-Dab^{20}-Ser^{21}-Leu/Ile^{22}$
Cichopeptin B	$CH_3-(CH_2)_n-CH=CH-(CH_2)_m-CH(OH)-CH_2-CO-Dhb^{1}-Pro^{2}-Ala^{3}-Ala^{4}-Ala^{5}-Ala^{6}-Val^{7}-Dhb^{8}-Gly^{9}-Val^{10}-Ile^{11}-Gly^{12}-Ala^{13}-Val^{14}-Ala^{15}-Val^{16}-Dhb^{17}-Thr^{18}-Ala^{19}-Dab^{20}-Ser^{21}-Val^{22}$
Corpeptin A	$CH_3-(CH_2)_6-CH(OH)-CH_2-CO-Dhb^{1}-Pro^{2}-Ala^{3}-Ala^{4}-Ala^{5}-Val^{6}-Val^{7}-Dhb^{8}-Hse^{9}-Val^{10}-Ile^{11}-Dhb^{12}-Ala^{13}-Ala^{14}-Ala^{15}-Val^{16}-Dhb^{17}-Thr^{18}-Ala^{19}-Dab^{20}-Ser^{21}-Ile^{22}$
Corpeptin B	$CH_3-(CH_2)_8-CH(OH)-CH_2-CO-Dhb^{1}-Pro^{2}-Ala^{3}-Ala^{4}-Ala^{5}-Val^{6}-Val^{7}-Dhb^{8}-Hse^{9}-Val^{10}-Ile^{11}-Dhb^{12}-Ala^{13}-Ala^{14}-Ala^{15}-Val^{16}-Dhb^{17}-Thr^{18}-Ala^{19}-Dab^{20}-Ser^{21}-Ile^{22}$
SP22A	$CH_3-(CH_2)_6-CH(OH)-CH_2-CO-Dhb^{1}-Pro^{2}-Val^{3}-Val^{4}-Ala^{5}-Ala^{6}-Val^{7}-Val^{8}-Dhb^{9}-Ala^{10}-Val^{11}-Ala^{12}-Ala^{13}-Dhb^{14}-Thr^{15}-Ser^{16}-Ala^{17}-Dhb^{18}-Ala^{19}-Dab^{20}-Dab^{21}-Tyr^{22}$
SP22B	$CH_3-(CH_2)_8-CH(OH)-CH_2-CO-Dhb^{1}-Pro^{2}-Val^{3}-Val^{4}-Ala^{5}-Ala^{6}-Val^{7}-Val^{8}-Dhb^{9}-Ala^{10}-Val^{11}-Ala^{12}-Ala^{13}-Dhb^{14}-Thr^{15}-Ser^{16}-Ala^{17}-Dhb^{18}-Ala^{19}-Dab^{20}-Dab^{21}-Tyr^{22}$
SP(SC)-1	$CH_3-(CH_2)_6-CH(OH)-CH_2-CO-Dhb^{1}-Pro^{2}-Val^{3}-Leu^{4}-Ala^{5}-Ala^{6}-Leu^{7}-Val^{8}-Dhp^{9}-Ala^{10}-Val^{11}-Ala^{12}-Ala^{13}-Dhb^{14}-Thr^{15}-Ser^{16}-Ala^{17}-Dhb^{18}-Ala^{19}-Dab^{20}-Dab^{21}-Tyr^{22}$
SP(SC)-2	$CH_3-(CH_2)_8-CH(OH)-CH_2-CO-Dhb^{1}-Pro^{2}-Val^{3}-Leu^{4}-Ala^{5}-Ala^{6}-Leu^{7}-Val^{8}-Dhb^{9}-Ala^{10}-Val^{11}-Ala^{12}-Ala^{13}-Dhb^{14}-Thr^{15}-Ser^{16}-Ala^{17}-Dhb^{18}-Ala^{19}-Dab^{20}-Dab^{21}-Tyr^{22}$
SP22phvA	$CH_3-(CH_2)_6-CH(OH)-CH_2-CO-Dhb^{1}-Pro^{2}-Val^{3}-Leu^{4}-Ala^{5}-Ala^{6}-Ala^{7}-Val^{8}-Dhb^{9}-Ala^{10}-Val^{11}-Ala^{12}-Ala^{13}-Dhb^{14}-Thr^{15}-Ser^{16}-Ala^{17}-Dhb^{18}-Ala^{19}-Dab^{20}-Dab^{21}-Tyr^{22}$
SP22phvB	$CH_3-(CH_2)_8-CH(OH)-CH_2-CO-Dhb^{1}-Pro^{2}-Val^{3}-Leu^{4}-Ala^{5}-Ala^{6}-Ala^{7}-Val^{8}-Dhb^{9}-Ala^{10}-Val^{11}-Ala^{12}-Ala^{13}-Dhb^{14}-Thr^{15}-Ser^{16}-Ala^{17}-Dhb^{18}-Ala^{19}-Dab^{20}-Dab^{21}-Tyr^{22}$
SP508A	$CH_3-(CH_2)_8-CH(OH)-CH_2-CO-Dhb^{1}-Pro^{2}-Val^{3}-Leu^{4}-Ala^{5}-Ala^{6}-Leu^{7}-Val^{8}-Ala^{9}-Ala^{10}-Val^{11}-Ala^{12}-Ala^{13}-Dhb^{14}-Thr^{15}-Ser^{16}-Ala^{17}-Dhb^{18}-Ala^{19}-Dab^{20}-Dab^{21}-Tyr^{22}$
SP508B	$CH_3-(CH_2)_{10}-CH(OH)-CH_2-CO-Dhb^{1}-Pro^{2}-Val^{3}-Leu^{4}-Ala^{5}-Ala^{6}-Leu^{7}-Val^{8}-Ala^{9}-Ala^{10}-Val^{11}-Ala^{12}-Ala^{13}-Dhb^{14}-Thr^{15}-Ser^{16}-Ala^{17}-Dhb^{18}-Ala^{19}-Dab^{20}-Dab^{21}-Tyr^{22}$

Supplementary Fig. 8: Structural comparison between R401 brassicapeptins and previously identified syringopeptins. Structures of brassicapeptins A-D, cichopeptins, corpeptins and other syringopeptins containing 22-amino-acid residues[44, 45, 46, 47]. Note that the proposed structures of the minor variants brassicapeptin C and D are putative and based on MS/MS fragmentation patterns. Cichopeptins comprise an unsaturated C12 fatty acid chain, however the location of the double bond could not be deduced. Hence a generic formula for the unsaturated lipid tail is given for cichopeptin A and B."

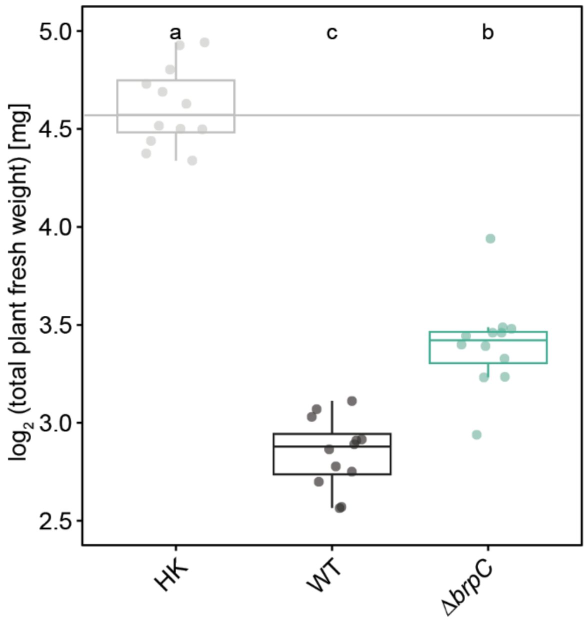

Supplementary Fig. 9: *brpC* contributes to R401 detrimental activity in agar-based gnotobiotic plant growth system. Log2-transformed fresh weight of *A. thaliana* plants (roots and shoot) grown on MS agar plates in the presence of heat-killed (HK), live wild-type (WT) or live Δ*brpC* R401 cells for 14 dpi. Letters indicate statistical significance as determined by Kruskal-Wallis followed by Dunn's post-hoc test; n = 12 plants. Boxplots show 25th percentile, median, and 75th percentile.

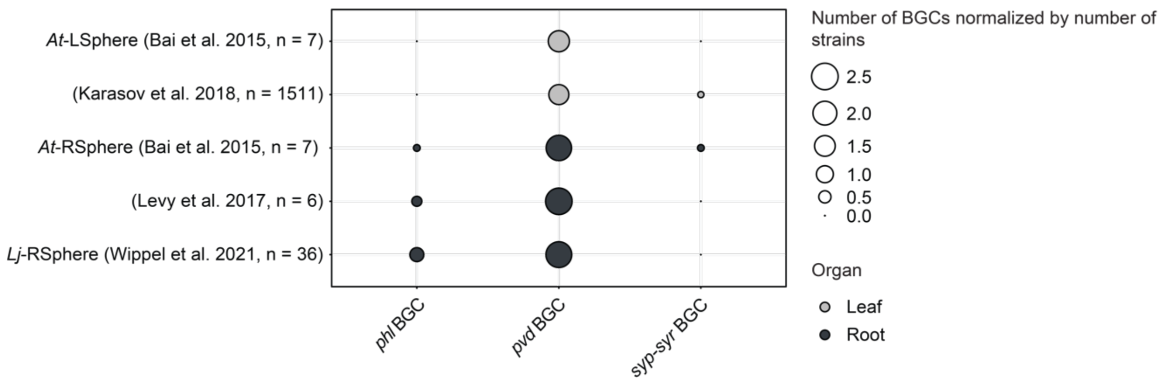

Supplementary Fig. 10: *Syp-syr* BGC is rare in genomes of plant-derived *Pseudomonas sp.* isolates. Using antiSMASH, we predicted BGCs for the genomes of *Pseudomonas sp.* isolates from different root and leaf derived culture collections. For each culture collection, the normalized number of *phl* BGCs (DAPG), *pvd* BGCs (pyoverdine), and syp-syr BGCs (syringo/brassica-peptin) are depicted. Dot size indicates the number of detected BGCs normalized by the number of tested strains.

Supplementary Notes

Supplementary Note 1: Structural characterisation of R401 brassicapeptins.

Structural characterisation of R401 brassicapeptins was achieved by cultivating 70 liters of axenically grown R401. The fermentation broth was extracted using the organic solvent ethyl acetate (EtOAc) extraction, which yielded 16.24 g. This crude extract was subjected to chromatographic purification, using reversed phase C18 column chromatography, followed by semipreparative high-performance liquid chromatography (HPLC). In that way, brassicapeptins A and B were isolated, both as a white amorphous powder. The molecular formula of brassicapeptins A and B were predicted using high-resolution electrospray ionization mass spectrometry (HR-ESI-MS). In the HR-ESI-MS spectrum of brassicapeptin A, two double-charged ion peaks, *i.e.*, $[M+2H]^{2+}$ at *m*/*z* 1027.1080 and $[M+Na+H]^{2+}$ at *m*/*z* 1038.0990, as well as a triple-charged ion peak $[M+3H]^{3+}$ at *m*/*z* 685.0747 (**Supplementary Fig. 7a**), suggesting a molecular formula of $C_{96}H_{161}N_{23}O_{26}$ and requiring 28 degrees of unsaturation. For brassicapeptin B, the molecular formula was suggested as $C_{94}H_{157}N_{23}O_{25}$ based on the two double-charged ion peaks $[M+2H]^{2+}$ at *m*/*z* 1005.0940 and $[M+Na+H]^{2+}$ at *m*/*z* 1016.0852, together with a triple-charged ion peak $[M+3H]^{3+}$ at *m*/*z* 670.4000 in the HR-ESI-MS spectrum (**Supplementary Fig. 7b**).

To fully resolve the structures, we performed nuclear magnetic resonance (NMR)-experiments of brassicapeptin A and Marfey's analysis of both derivatives, *i.e.*, brassicapeptin A and B. The ^{1}H NMR and Heteronuclear Single Quantum Coherence (HSQC) spectrum displayed 22 amide protons, which resonated from δ_H 7.30 to δ_H 9.40 and 18 α protons that resonated from δ_H 3.70 to δ_H 4.50 (**Supplementary Table 6**). A correlation from a methylene group at δ_H 2.85 and 2.77 (Dab-Hγ) to amide protons at δ_H 7.68 (Dab-NH_2, 2H, overlapped) from the ^{1}H-^{1}H Correlated Spectroscopy (COSY) spectrum indicated a terminal amide group (Dab-γ) belonging to a diaminobutyric acid (Dab) residue, which was confirmed by Total Correlation Spectroscopy (TOCSY) correlation from Dab-NH (δ_H 9.20) to Dab-Hα (δ_H 3.82), Dab-Hβ (δ_H 2.32 and 2.02), Dab-Hγ, and Dab-NH_2 (**Fig. 4d** and **Supplementary Table 6**). A dehydroalanine (Dha) residue was assigned based on the Heteronuclear Multiple Bond Correlation (HMBC) correlations from δ_H 5.97 and 5.52 (Dha-Hβ) to δ_C 135.32 (Dha-Cα) and δ_C 163.74 (Dha-CO) (**Fig. 4d** and **Supplementary Table 6**). Three 2,3-dehydroaminobutyric acid (Dhb) residues were assigned based on clear COSY correlations between three olefinic protons (Dhb-Hβ) and three methyl groups (Dhb-Hγ) (i.e., δ_H 6.35/δ_H 1.61, δ_H 6.25/δ_H 1.67, and δ_H 6.07/δ_H 1.66), as well as HMBC correlations from the olefinic proton of Dhb-Hβ to carbon of Dhb-Cα and Dhb carbonyl (*i.e.*, from δ_H 6.35 to δ_C 130.47 and 164.11, from δ_H 6.25 to δ_C 130.89 and 164.15, and from δ_H 6.07 to δ_C 131.51 and δ_C 165.93), and from the methyl group of Dhb-Hγ to carbon of Dhb-Cβ and Dhb-Cα (*i.e.*, from δ_H 1.61 to δ_C 127.82 and δ_C 130.47; from δ_H 1.67 to δ_C 127.02 and δ_C 130.89; from δ_H 1.66 to δ_C 124.14 and δ_C 131.51) (**Fig. 4d** and **Supplementary Table 6**). A threonine (Thr) residue was identified from the TOCSY correlations between Thr-NH (δ_H 7.84)/Thr-Hα (δ_H 4.16), Thr-NH/Thr-Hβ (δ_H 4.90) and Thr-Hβ/Thr-Hγ (δ_H 1.16) (**Fig. 4d** and **Supplementary Table 6**). The presence of a serine (Ser) residue was verified based on the COSY correlations between Ser-Hβ (δ_H 3.73 and 3.87)/Ser-Hα (δ_H 4.23), together with the TOCSY correlations between Ser-NH (δ_H 7.45)/Ser-Hα and Ser-NH/Ser-Hβ (**Fig. 4d** and **Supplementary Table 6**). In addition, we also clarified the presence of a homoserine (Hse) residue from the COSY correlations between Hse-Hα (δ_H 4.39)/Hse-Hβ (δ_H 1.73 and 1.84) and Hse-Hβ/Hse-Hγ (δ_H 3.41), as well as TOCSY correlations from Hse-NH (δ_H 7.56) to Hse-Hα, Hse-Hβ and Hse-Hγ (**Fig. 4d** and **Supplementary Table 6**). Two leucine (Leu) residues were verified based on the TOCSY correlations between Leu-NH/Leu-Hα and Leu-NH/Leu-Hβ (*i.e.*, δ_H 7.54/δ_H 4.15, δ_H 7.54/δ_H 1.49, δ_H 7.90/δ_H 4.36 and δ_H 7.90/δ_H 1.13), together with HMBC correlations from Leu-Hβ to Leu-Cγ, Leu-Cδ and Leu-Cδ' (*i.e.*, from δ_H 1.49 to δ_C 24.05, 21.22, 22.89, and from δ_H 1.32 and 1.13 to δ_C 36.25, 11.43 and 14.44) (**Fig. 4d** and **Supplementary Table 6**). Two isoleucine (Ile) residues were identified based on the TOCSY correlations from Ile-NH to Ile-Hα, Ile-Hβ and Ile-Hγ' (*i.e.*, from δ_H 7.75 to δ_H 4.28, δ_H 1.86 and δ_H 0.86 and from δ_H 7.39 to δ_H 4.42, δ_H 1.89 and 0.80), as well as

COSY correlations from Ile-Hγ to Ile-Hβ and Ile-Hδ (i.e., from δ_H 1.41 and 1.13 to δ_H 1.86 and δ_H 0.89, and from δ_H 1.47 and 1.05 to δ_H 1.89 and δ_H 0.84) (**Fig. 4d** and **Supplementary Table 6**). Three valine (Val) residues were assigned based on TOCSY correlations from Val-NH to Val-Hα and Val-Hβ (*i.e.*, from δ_H 7.66 to δ_H 4.19 and δ_H 1.99, from δ_H 7.71 to δ_H 4.25 and δ_H 2.03, and from δ_H 7.83 to δ_H 4.16 and δ_H 2.06), together with HMBC correlations from Val-Hγ and Val-Hγ' to Val-Cβ (*i.e.*, from δ_H 0.79 and 0.83 to δ_C 29.77, from δ_H 0.81 and 0.83 to δ_C 30.22, and from δ_H 0.91 and 0.93 to δ_C 30.05) (**Fig. 4d** and **Supplementary Table 6**). In addition, we also clarified the presence of seven alanine (Ala) residues from the TOCSY correlations from Ala-NH to Ala-Hα and Ala-Hβ (*i.e.*, from δ_H 8.34 to δ_H 4.32 and δ_H 1.28, from δ_H 8.26 to δ_H 4.20 and δ_H 1.27, from δ_H 8.02 to δ_H 4.24 and δ_H 1.23, from δ_H 7.96 to δ_H 3.99 and δ_H 1.24, from δ_H 7.95 to δ_H 4.33 and δ_H 1.22, from δ_H 7.91 to δ_H 4.13 and δ_H 1.29, and from δ_H 7.65 to δ_H 4.24 and δ_H 1.23) (**Fig. 4d** and **Supplementary Table 6**). The above 1D and 2D NMR data revealed 22 amino acid residues of Brassicapeptin A, including one diaminobutyric acid (Dab), one dehydroalanine (Dha), three 2,3-dehydroaminobutyric acids (Dhb), one threonine (Thr), one serine (Ser), one homoserine (Hse), two leucines (Leu), two isoleucines (Ile), three valines (Val), and seven alanines (Ala). Additionally, four methylene groups at δ_C 34.82 (δ_H 2.30 and 2.26), δ_C 30.77 (δ_H 1.27), δ_C 24.49 (δ_H 1.53) and δ_C 21.83 (δ_H 1.28) and one methyl at δ_C 13.78 (δ_H 0.86) were observed from the HSQC spectrum, together with another carbonyl group at δ_C 172.58 detected from the ^{13}C NMR spectrum (**Fig. 4d** and **Supplementary Table 6**). These signals were attributed to a fatty acid (FA) chain consisting of six carbons, thereby including a carbonyl group. This was based on TOCSY correlations from FA-H_2 (δ_H 2.30 and δ_H 2.26) to FA-H_3 (δ_H 1.53), FA-H_4 (δ_H 1.27), FA-H_5 (δ_H 1.28) and FA-H_6 (δ_H 0.86), as well as HMBC correlations from FA-H_2 to FA-CO (δ_C 172.58), FA-C_4 (δ_C 30.77) and FA-C_3 (δ_C 24.49), from FA-H_4 to FA-C_3 and FA-C_5 (δ_C 21.83), and from both FA-H_3 and FA-H_6 (δ_H 0.86) to FA-C_5 (**Fig. 4d** and **Supplementary Table 6**). The fatty acid chain and the amino acid residues accounted for 27 of the 28 degrees of unsaturation indicated by the molecular formula. Therefore, it was concluded that brassicapeptin A possesses an additional ring.

The connectivity and the sequence of the amino acids for Brassicapeptin A was established by HMBC and Rotating-frame Nuclear Overhauser Effect Spectroscopy (ROESY) NMR data, along with the high-resolution electrospray ionization tandem mass spectrometry (HR-ESI-MS/MS) data. The fatty acid chain was assigned to be linked with a Dhb residue (Dhb_1) based on HMBC correlation from Dhb_1-NH (δ_H 9.38) to FA-CO, which can be confirmed by the ROESY correlation between Dhb_1-NH and FA-H_2 (**Fig. 4d** and **Supplementary Table 6**). The Dhb_1 residue was further supported to be linked with a peptide fragment of Ala_2-Ala_3-Leu_4-Ala_5 by ROESY correlations between Dhb_1-NH/Ala_2-NH (δ_H 8.26), Dhb_1-Hβ (δ_H 6.07)/Ala_2-NH, Ala_2-NH/Ala_3-NH (δ_H 7.91), Ala_3-Hβ (δ_H 1.29)/Leu_4-NH (δ_H 7.54), Leu_4-Hα (δ_H 4.15)/Ala_5-NH (δ_H 7.65), as well as by HMBC correlation from Ala_2-NH to Dhb_1-CO (δ_C 165.93), from Ala_3-NH to Ala_2-CO (δ_C 172.96), from Leu_4-NH to Ala_3-CO (δ_C 172.25), and from Ala_5-NH to Leu_4-CO (δ_C 171.68) (**Fig. 4d** and **Supplementary Table 6**). Thus, a FA-Dhb_1-Ala_2-Ala_3-Leu_4-Ala_5 part was elucidated, which was also supported by the MS/MS fragments b_1-b_4 (**Fig. 4a)**. Key ROESY correlations were observed between Ala_5-Hα (δ_H 4.24)/Val_6-NH (δ_H 7.66), Val_6-Hα (δ_H 4.19)/Ile_7-NH (δ_H 7.75), Ile_7-NH/Dhb_8-NH (δ_H 9.14), Dhb_8-NH/Hse_9-NH (δ_H 7.56), Hse_9-Hα (δ_H 4.39)/Val_{10}-NH (δ_H 7.71), Val_{10}-Hα (δ_H 4.25)/Leu_{11}-NH (δ_H 7.90), and Leu_{11}-NH/Dha_{12}-NH (δ_H 9.12), as well as key HMBC correlations of NH signals of Val_6, Ile_7, Dhb_8, Hse_9, Val_{10}, Leu_{11} and Dha_{12}, respectively, to their neighboring carbonyls of Ala_5 (δ_C 172.21), Val_6 (δ_C 171.32), Ile_7 (δ_C 170.46), Dhb_8 (δ_C 164.11), Hse_9 (δ_C 171.38), Val_{10} (δ_C 171.42), and Leu_{11} (δ_C 170.68), which correspond to the peptide fragment of Val_6-Ile_7-Dhb_8-Hse_9-Val_{10}-Leu_{11}-Dha_{12} (**Fig. 4d** and **Supplementary Table 6**). This assignment was also evident from the MS/MS fragments b_5-b_{11} and y_7-y_{14} (**Fig. 4a**). Further ROESY correlations between Dha_{12}-Hβ (δ_H 5.97 and 5.52)/Ala_{13}-NH (δ_H 8.34), Ala_{13}-Hα (δ_H 4.32)/Ala_{14}-NH (δ_H 8.02), Ala_{14}-Hα (δ_H 4.24)/Ala_{15}-NH (δ_H 7.95), and Ala_{15}-Hα (δ_H 4.33)/Val_{16}-NH (δ_H 7.83), in association with the HMBC correlations from Val_{16}-NH to Ala_{15}-CO (δ_C 172.89), from Ala_{15}-NH to Ala_{14}-CO (δ_C 172.04), from Ala_{14}-NH to Ala_{13}-CO (δ_C 171.94), and from Ala_{13}-NH to Dha_{12}-CO (δ_C 163.74), established another partial peptide sequence of Dha_{12}-Ala_{13}-Ala_{14}-Ala_{15}-Val_{16}, which was in

agreement with the MS/MS fragments y_3-y_8 (**Fig. 4a,d** and **Supplementary Table 6**). These data corroborated the linear chain of brassicapeptin A as FA-Dhb_1-Ala_2-Ala_3-Leu_4-Ala_5-Val_6-Ile_7-Dhb_8-Hse_9-Val_{10}-Leu_{11}-Dha_{12}-Ala_{13}-Ala_{14}-Ala_{15}-Val_{16}. Similarly, the observed ROESY correlation of Ile_{22}-NH (δ_H 7.39)/Ser_{21}-Hα (δ_H 4.23), Ser_{21}-NH (δ_H 7.45)/Dab_{20}-Hα (δ_H 3.82) and Dab_{20}-NH (δ_H 9.20)/Ala_{19}-Hα (δ_H 3.99) suggested the fragment of Ala_{19}-Dab_{20}-Ser_{21}-Ile_{22}, which was evident from the MS/MS fragment y_1 (**Fig. 4a,d** and **Supplementary Table 6**). Further ROESY correlations between Dhb_{18}-NH (δ_H 9.27)/Ala_{19}-Hα, and Dhb_{18}-NH/Thr_{17}-NH (δ_H 7.84), along with the HMBC correlations from Dhb_{18}-NH to Thr_{17}-CO (δ_C 170.53), established the partial structure of Thr_{17}-Dhb_{18}-Ala_{19}, which was confirmed by the fragments y_2 and y_3 (**Fig. 4a,d** and **Supplementary Table 6**). Moreover, the ROESY correlation observed for Ile_{22}-Hα (δ_H 4.42)/Thr_{17}-Hγ (δ_H 1.16), in addition to the HMBC correlation from Thr_{17}-Hβ (δ_H 4.90) to Ile_{22}-CO (δ_C 169.45), established the nature of the cyclization of brassicapeptin A via the connection of the Thr_{17} unit with Ile_{22}, fulfilling the degrees of unsaturation and further supported by the detected MS/MS fragment y_3 (**Fig. 4a,d** and **Supplementary Table 6**). Thus, the cyclic substructure was established as *cyclo*(Thr_{17}-Dhb_{18}-Ala_{19}-Dab_{20}-Ser_{21}-Ile_{22}). Finally, the connectivity of the cyclic part and the linear chain was established through the HMBC correlation from Thr_{17}-NH to Val_{16}-CO (δ_C 172.86), which was further corroborated by the fragments y_3 and y_4 from the MS/MS data (**Fig. 4a,d** and **Supplementary Table 6**). Hence, the planar structure of brassicapeptin A was elucidated. Comparison of the HR-ESI-MS/MS fragments of brassicapeptins A and B revealed the high similarity from b_1 to b_7 and from y_1-y_{10}, except for the differences from b_7 to b_{11} and from y_{11}-y_{14} (**Fig. 4a,b**). These differences indicated that the Hse_9 residue present in brassicapeptin A was replaced by a Gly residue (**Fig. 4c**). Thus, the planar structure of brassicapeptin B was elucidated as FA-Dhb_1-Ala_2-Ala_3-Leu_4-Ala_5-Val_6-Ile_7-Dhb_8-Gly_9-Val_{10}-Leu_{11}-Dha_{12}-Ala_{13}-Ala_{14}-Ala_{15}-Val_{16}-*cyclo*(Thr_{17}-Dhb_{18}-Ala_{19}-Dab_{20}-Ser_{21}-Ile_{22}). We were not able to get the NMR data of brassicapeptin B due to the limited amount of sample.

Marfey's analysis and ROESY data was used to determine the absolute configuration. Brassicapeptins A and B were first hydrolyzed, followed by chemical derivatization with N_α-(2,4-dinitro-5-fluorophenyl)-L-valinamide (L-FDVA, Marfey's reagent), then comparing the retention time with the reference substrates using UPLC-MS analysis (**Supplementary Fig. 7j**). Marfey's analysis revealed that the homoserine residue (Hse_9), serine residue (Ser_{21}) and valine residues (Val_6, Val_{10}, and Val_{16}) were present in D-configuration, whereas the diaminobutyric acid residue (Dab_{20}) and threonine residue (Thr_{17}) were present in L-configuration (**Supplementary Fig. 7j**). The observed ROESY correlation for Thr_{17}-Hα/Thr_{17}-Hγ and Ile_{22}-Hα /Thr_{17}-Hγ indicated the R-configuration for Thr_{17}-Cβ and D-configuration for Ile_{22} (**Fig. 4d** and **Supplementary Table 6**). The Ile_7 was further assigned as L-configuration due to the fact that a mixture of both, D- and L-configuration of isoleucines were observed from Marfey's analysis (**Supplementary Fig. 7j**). Further detected ROESY correlations between Ala_{19}-Hβ/Thr_{17}-Hβ and Ala_{19}-Hβ/Dab_{20}-Hγ allowed the assignment of L-configuration for Ala_{19} (**Fig. 4d** and **Supplementary Table 6**). The ROESY correlations between Ala_{14}-Hα/Ala_{15}-Hα, Ala_2-Hβ/Leu_4-Hα and Ala_3-Hβ/Leu_4-Hα suggested the same configuration for Ala_{14} and Ala_{15}, same configuration for Ala_2 and Ala_3 and different configuration for Ala_3 and Leu_4. Additionally, a 2:5 ratio of L- and D-alanine was observed, indicating the D-configuration of Ala_2, Ala_3, Ala_{14} and Ala_{15} and L-configuration of Leu_4 (**Fig. 4d**, **Supplementary Table 6** and **Supplementary Fig. 7j**). Marfey's analysis also indicated the presence of both, L- and D-leucine, which allowed us to assign the D-configuration for Leu_{11} (**Supplementary Fig. 8j**). Additional ROESY correlations observed between Ala_{13}-Hα/Leu_{11}-Hδ' allowed the assignment of a L-configuration for Ala_{13} (**Fig. 4d** and **Supplementary Table 6**). The Ala_5 was then deduced as D-configuration from the observed 2:5 ratio of L- and D-alanine. The same absolute configuration of brassicapeptin B was established based on the same results of the Marfey's analysis, except the Hse_9 residue was replaced by a glycine (Gly) residue (**Fig. 4d** and **Supplementary Fig. 7j**). Thus, the structure of brassicapeptins A and B were elucidated as shown (**Fig. 4c)**.

Besides brassicapeptin A and B, another two minor derivatives (*i.e.*, brassicapeptins C and D) were also elucidated based on the HR-ESI-MS/MS data. In the HR-ESI-MS spectrum of brassicapeptin C, two double-charged ion peaks at at *m*/*z* 1020.1176 $[M+2H]^{2+}$ and

1031.1058 $[M+Na+H]^{2+}$ were observed (**Supplementary Fig. 7k**), suggesting a molecular formula of $C_{95}H_{159}N_{23}O_{26}$. The HR-ESI-MS/MS data of brassicapeptin C revealed high similarity to brassicapeptin A (**Supplementary Fig. 7k** and **Supplementary Table 7**). The fragments b_1-b_{10} were similar, while the fragments y_1-y_{11} exhibited a 14 Da difference to brassicapeptin A. These differences suggested that the amino acid residues from position 19 to 22 of the cyclic substructure in brassicapeptin C were different. A proposed sequence of Gly_{19}-Dab_{20}-Ser_{21}-Ile_{22} was supposed based on the 14 Da difference. The molecular formula of brassicapeptin D was suggested to be $C_{95}H_{159}N_{23}O_{26}$, which is identical to brassicapeptin C, based on the two double-charged ion peaks $[M+2H]^{2+}$ at *m/z* 1020.0997 and $[M+Na+H]^{2+}$ at *m/z* 1031.0908 (**Supplementary Fig. 7l**). However, the fragments y_1-y_{11} of brassicapeptin D were similar, while the fragments b_1-b_{10} exhibited a 14 Da difference, when compared the HR-ESI-MS/MS data of brassicapeptin A (**Supplementary Fig. 7l** and **Supplementary Table 7**). This suggests the same amino acid residues from position 3 to 22 (Ala_3-Leu_4-Ala_5-Val_6-Ile_7-Dhb_8-Gly_9-Val_{10}-Leu_{11}-Dha_{12}-Ala_{13}-Ala_{14}-Ala_{15}-Val_{16}-*cyclo*(Thr_{17}-Dhb_{18}-Ala_{19}-Dab_{20}-Ser_{21}-Ile_{22}) except for the fatty acid chain and the first two amino acid residues, which supposed to be $C_6H_{11}O$-Dhb_1-Gly_2 based on the 14 Da difference. We were not able to assign the substructure of the amino acid residues from position 19 to 22 and the first two amino acid residues for brassicapeptin D based on NMR data or Marfey's analysis, since we could not isolate these minor variants. Brassicapeptins A-D are new cyclic lipopeptides containing 22 amino acid residues, which show similarity to previously described syringopeptins (**Supplementary Fig. 8**)
