# Supplementary material for: Physiochemical interaction between osmotic stress and a bacterial exometabolite promotes plant disease
Source: Nat Commun. 2024 May 28;15:4438. doi: 10.1038/s41467-024-48517-5 (PMC11133316; doi:10.1038/s41467-024-48517-5)
Supplement: Supplementary file 3 — Description of Additional Supplementary Files [file 41467_2024_48517_MOESM3_ESM.pdf]

Description of Additional Supplementary Files:

Supplementary Data 1: A. thaliana DEGs in roots and shoots in response to R401 and/or NaCl stress

Supplementary Data 2: AntiSMASH-based prediction of brassicapeptin

Supplementary Data 3: Minimal inhibitory concentrations of R401 brassicapeptin A

Supplementary Data 4: Primers used in this study

Supplementary Data 5: Microorganisms used in this study

Supplementary Data 6: NMR data

Supplementary Data 7: MS/MS data
