# Supplementary material for: Physiochemical interaction between osmotic stress and a bacterial exometabolite promotes plant disease
Source: Nat Commun. 2024 May 28;15:4438. doi: 10.1038/s41467-024-48517-5 (PMC11133316; doi:10.1038/s41467-024-48517-5)
Supplement: Supplementary file 11 — Reporting Summary [file 41467_2024_48517_MOESM11_ESM.pdf]

nature portfolio

Corresponding author(s): Stéphane Hacquard

Last updated by author(s): Apr 29, 2024

Reporting Summary

Nature Portfolio wishes to improve the reproducibility of the work that we publish. This form provides structure for consistency and transparency in reporting. For further information on Nature Portfolio policies, see our Editorial Policies and the Editorial Policy Checklist.

Statistics

For all statistical analyses, confirm that the following items are present in the figure legend, table legend, main text, or Methods section.

n/a	Confirmed	
☐	☒	The exact sample size (*n*) for each experimental group/condition, given as a discrete number and unit of measurement
☐	☒	A statement on whether measurements were taken from distinct samples or whether the same sample was measured repeatedly
☐	☒	The statistical test(s) used AND whether they are one- or two-sided<br>*Only common tests should be described solely by name; describe more complex techniques in the Methods section.*
☒	☐	A description of all covariates tested
☐	☒	A description of any assumptions or corrections, such as tests of normality and adjustment for multiple comparisons
☐	☒	A full description of the statistical parameters including central tendency (e.g. means) or other basic estimates (e.g. regression coefficient) AND variation (e.g. standard deviation) or associated estimates of uncertainty (e.g. confidence intervals)
☐	☒	For null hypothesis testing, the test statistic (e.g. *F*, *t*, *r*) with confidence intervals, effect sizes, degrees of freedom and *P* value noted<br>*Give P values as exact values whenever suitable.*
☒	☐	For Bayesian analysis, information on the choice of priors and Markov chain Monte Carlo settings
☒	☐	For hierarchical and complex designs, identification of the appropriate level for tests and full reporting of outcomes
☒	☐	Estimates of effect sizes (e.g. Cohen's *d*, Pearson's *r*), indicating how they were calculated

Our web collection on statistics for biologists contains articles on many of the points above.

Software and code

Policy information about availability of computer code

Data collection	No software was used to collect data
Data analysis	Differential gene expression was performed using DESeq2. All statistical analyses were conducted in R 4.1.2. Data visualisation was conducted using the ggplot2 package (as part of the Tidyverse) or the ComplexHeatmap package. As nonparametric tests, Kruskal-Wallis followed by Dunn's post-hoc test and Benjamini-Hochberg (BH) adjustment for multiple comparisons from the PMCMRplus package were used. The respective statistical tests are indicated in each figure description. Significance was indicated by significance group ($p \leq 0.05$). No statistical methods were used to pre-determine sample sizes. Halo size quantification in modified Burkholder experiments, and root length measurements were performed blinded using the Fiji package of ImageJ. Colony counts of R401 were performed blinded. RNA sequencing data processed as described above and further analyzed and visualized as previously described6. GO Term enrichment was conducted as indicted in the respective figure description or results section. Figures were assembled in Adobe Illustrator.

For manuscripts utilizing custom algorithms or software that are central to the research but not yet described in published literature, software must be made available to editors and reviewers. We strongly encourage code deposition in a community repository (e.g. GitHub). See the Nature Portfolio guidelines for submitting code & software for further information.

Data

Policy information about availability of data

All manuscripts must include a data availability statement. This statement should provide the following information, where applicable:

- Accession codes, unique identifiers, or web links for publicly available datasets
- A description of any restrictions on data availability
- For clinical datasets or third party data, please ensure that the statement adheres to our policy

Data availability
RNAseq read data are available at GEO accession: GSE242479. A Source Data file is provided with this paper and all included data is also accessible from the link in the code availability section. A spreadsheet with all exact p-values is provided in the Source Data file.

Code availability
All codes and respective data generated for this study are available at https://github.com/scriptsFG/Getzke-Wang-et-al-2023.git

Research involving human participants, their data, or biological material

Policy information about studies with human participants or human data. See also policy information about sex, gender (identity/presentation), and sexual orientation and race, ethnicity and racism.

Reporting on sex and gender	Not applicable
Reporting on race, ethnicity, or other socially relevant groupings	Not applicable
Population characteristics	Not applicable
Recruitment	Not applicable
Ethics oversight	Not applicable

Note that full information on the approval of the study protocol must also be provided in the manuscript.

Field-specific reporting

Please select the one below that is the best fit for your research. If you are not sure, read the appropriate sections before making your selection.

☒ Life sciences ☐ Behavioural & social sciences ☐ Ecological, evolutionary & environmental sciences

For a reference copy of the document with all sections, see nature.com/documents/nr-reporting-summary-flat.pdf

Life sciences study design

All studies must disclose on these points even when the disclosure is negative.

Sample size	No statistical methods were used to pre-determine sample sizes. The total number of samples used for each condition is always > 10. Individual datapoints are shown in each boxplots.
Data exclusions	No samples were excluded from the analyses
Replication	RNAseq experiment was performed three times independently. All other experiments include data from at least two independent experiments, each containing at least 5 technical replicates.
Randomization	All agar plates and box for flowpots experiment was distributed randomly into the growth chambers, and randomly shuffled every three days to minimize location-based effects. Samples were collected randomly for each condition in all experiments.
Blinding	Numbers were used to avoiding condition identification during sampling.

Reporting for specific materials, systems and methods

We require information from authors about some types of materials, experimental systems and methods used in many studies. Here, indicate whether each material, system or method listed is relevant to your study. If you are not sure if a list item applies to your research, read the appropriate section before selecting a response.

Materials & experimental systems

n/a	Involved in the study
[x]	[ ] Antibodies
[x]	[ ] Eukaryotic cell lines
[x]	[ ] Palaeontology and archaeology
[x]	[ ] Animals and other organisms
[x]	[ ] Clinical data
[x]	[ ] Dual use research of concern
[ ]	[x] Plants

Methods

n/a	Involved in the study
[x]	[ ] ChIP-seq
[x]	[ ] Flow cytometry
[x]	[ ] MRI-based neuroimaging

Dual use research of concern

Policy information about dual use research of concern

Hazards

Could the accidental, deliberate or reckless misuse of agents or technologies generated in the work, or the application of information presented in the manuscript, pose a threat to:

No	Yes
[x]	[ ] Public health
[x]	[ ] National security
[x]	[ ] Crops and/or livestock
[x]	[ ] Ecosystems
[x]	[ ] Any other significant area

Experiments of concern

Does the work involve any of these experiments of concern:

No	Yes
[x]	[ ] Demonstrate how to render a vaccine ineffective
[x]	[ ] Confer resistance to therapeutically useful antibiotics or antiviral agents
[x]	[ ] Enhance the virulence of a pathogen or render a nonpathogen virulent
[x]	[ ] Increase transmissibility of a pathogen
[x]	[ ] Alter the host range of a pathogen
[x]	[ ] Enable evasion of diagnostic/detection modalities
[x]	[ ] Enable the weaponization of a biological agent or toxin
[x]	[ ] Any other potentially harmful combination of experiments and agents

Plants

Seed stocks	A. thaliana ecotype Columbia-0 (Col-0), Lotus japonicus ecotype Gifu B-129, and Solanum lycopersicum cv. Micro-Tom were used as wild-types. A. thaliana rbohd mutant contains a dSpm transposon in the fifth exon of AtrbohD (AT5G47910, Torres et al. 2002)
Novel plant genotypes	None
Authentication	not applicable
